# Noise-Limited Failure of OGY Chaos Control in Regulating Monosynaptic Reflex Variability in the In Vivo Cat Spinal Cord

**DOI:** 10.3390/neurosci7010018

**Published:** 2026-02-02

**Authors:** Elias Manjarrez, Ignacio Méndez-Balbuena, Saul M. Dominguez-Nicolas, Oscar Arias-Carrión

**Affiliations:** 1 Instituto de Fisiología, Benemérita Universidad Autónoma de Puebla, 14 Sur 6301, Col. San Manuel, Apartado Postal 406, Puebla 72570, Mexico; 2 Facultad de Psicología, Benemérita Universidad Autónoma de Puebla, 3 Oriente 1413, Barrio de Analco, Puebla 72500, Mexico; ignacio.mendez@correo.buap.mx; 3 Centro de Micro y Nanotecnología, Universidad Veracruzana, Calzada Ruiz Cortines 455, Veracruz 94294, Mexico; saudominguez@uv.mx; 4 División de Neurociencias, Clínica, Instituto Nacional de Rehabilitación Luis Guillermo Ibarra Ibarra, Mexico City 14389, Mexico; 5 Escuela de Medicina y Ciencias de la Salud, Tecnológico de Monterrey, Mexico City 14380, Mexico

**Keywords:** spinal cord physiology, reflex, monosynaptic, chaos theory, systems control engineering, neuronal noise, cats

## Abstract

Monosynaptic reflexes (MSRs) elicited by constant-intensity group I afferent stimulation exhibit marked amplitude variability, commonly attributed to stochastic presynaptic modulation and dynamic postsynaptic excitability. Here, we tested whether this variability could be attenuated using the Ott–Grebogi–Yorke (OGY) chaos–control algorithm, which stabilizes unstable periodic orbits in low-dimensional nonlinear systems. In spinalized, anesthetized cats, real-time implementation of the OGY method failed to reduce MSR amplitude variability, as quantified by the coefficient of variation, and the return map structure showed no evidence of orbit stabilization. These negative results contrast with successful applications of OGY control in physical systems, cardiac tissue, hippocampal slices, and stochastic neuronal models. We interpret this failure in the context of the intense, ongoing synaptic bombardment characteristic of dorsal horn circuitry, which likely obscures or destroys the low-dimensional geometric structure required for OGY-based control. Our findings delineate a fundamental limit to classical chaos–control algorithms in intact neural circuits and highlight the need for control strategies explicitly robust to high dimensionality and physiological noise.

## 1. Introduction

Biological systems often exhibit complex nonlinear dynamics that can appear irregular or “chaotic” without necessarily implying dysfunction. In this sense, chaos and stabilization may coexist in living biological systems, reflecting the balance between flexibility and regulation required for adaptive function. In neural systems, particularly in the brain, activity may operate near a regime between excessive order and excessive instability, sometimes referred to as an “edge-of-chaos” or near-critical state [1], which has been proposed to support flexible and adaptive information processing. Stabilization in this context does not imply eliminating variability, but rather regulating it within functional bounds through physiological feedback mechanisms such as homeostasis and activity-dependent plasticity. This perspective has motivated growing interest in applying dynamical systems and control concepts to biological circuits.

Chaos pervades the dynamics of many biological and physical systems. Nevertheless, the possibility of controlling chaos emerged only when Ott, Grebogi, and Yorke made the counterintuitive prediction that unstable periodic orbits, normally buried within a chaotic attractor, could be selectively stabilized through vanishingly small, precisely timed perturbations [2]. This now-classic Ott–Grebogi–Yorke (OGY) method reframed chaos not as a barrier to control but as a reservoir of latent structure. Rather than redesigning a system or suppressing its intrinsic nonlinearities, OGY exploits the geometry of its dynamics, using delay-coordinate reconstruction to identify local linearizations around a targeted orbit and nudging the system back toward stability through minimal parametric adjustments. Its elegance lies in its parsimony: no detailed mechanistic model is required; only the capacity to track the system’s state and modulate one experimentally accessible parameter is needed.

Early demonstrations of OGY control in physical systems established its feasibility under real-world noise and parameter drift. Ditto and colleagues famously stabilized period-1 and period-2 orbits embedded in the chaotic vibrations of a driven magnetoelastic ribbon, even switching among them at will [3]. Similar success followed in optical systems, where OGY-based interventions tamed the chaotic output of lasers [4]. Taken together, these experiments fostered optimism that chaos control might serve as a broadly applicable tool for engineering nonlinear systems.

The biological sciences soon became fertile ground for this optimism. Garfinkel et al. showed that OGY-style perturbations could regularize chaotic dynamics in cardiac tissue by suppressing arrhythmogenic activity through subtle timing adjustments [5]. Schiff and colleagues extended this achievement to the nervous system: in rat hippocampal slices, they identified unstable periodic orbits governing spontaneous CA3 bursting and stabilized them using minute electrical stimuli [6]. These experiments suggested that even complex neural networks could harbor low-dimensional structures amenable to chaos control—a perspective that catalyzed efforts to apply dynamical systems methods to the pathological rhythms of the heart and brain.

However, the biological implementations of OGY also revealed ambiguities. Christini and Collins demonstrated that OGY-like stabilization could emerge in stochastic systems that lack deterministic chaos [7].

Recent computational studies continue to demonstrate how nonlinear dynamical methods can provide mechanistic insights into complex biological systems. For example, Joshi (2024) [8] introduced a mathematical model to investigate COVID-19 transmission dynamics while incorporating neurodegeneration and memory-trace effects, using fixed-point analysis and numerical experiments to examine the impact of biological parameters on system behavior. Such theoretical approaches highlight the broader applicability of dynamical systems concepts, such as fixed points, stability, and parameter dependence, in modeling and interpreting complex physiological processes.

In a FitzHugh-Nagumo model driven by noise, proportional perturbation feedback reproduced the signature “control” effects previously attributed to deterministic unstable periodic orbits. Their findings challenged the assumption that successful stabilization necessarily implies deterministic chaos, suggesting that OGY-like methods act more as noise-shaping filters than as orbit-stabilizing mechanisms.

Despite this conceptual tension, recent theoretical and applied studies have continued to deploy OGY control across disparate domains, from infectious disease dynamics [9] to discrete prey-predator interactions exhibiting rich nonlinear behavior [10,11,12,13]. Across these contexts, OGY appears surprisingly tolerant of noise, often in defiance of the original theoretical caveat: that stochastic fluctuations could prevent the system from remaining within the narrow basin surrounding an unstable periodic orbit [2].

A critical observation emerges from surveying this literature: nearly all successful biological applications of OGY have occurred in highly reduced preparations: in vitro cardiac monolayers, isolated hippocampal circuits, or simplified stochastic models. These systems, by virtue of their low dimensionality and limited afferent bombardment, possess the dynamical “quietude” necessary for unstable periodic orbits to be meaningfully identified and stabilized. Whether OGY control remains viable in intact, high-dimensional neural circuits that are continuously driven by sensory inflow, local circuit noise, neuromodulatory fluctuations, and correlated network activity remains unknown.

The spinal monosynaptic reflex (MSR) pathway provides an especially tractable system for examining this question. In the spinalized cat—an archetypal preparation for studying reflex physiology—successive MSRs evoked by constant-strength group I stimulation fluctuate markedly in amplitude, largely due to stochastic presynaptic inhibition and dynamic variations in motoneuron excitability [14,15]. These fluctuations are not merely measurement noise but reflect ongoing dorsal horn activity that shapes the excitability of the monosynaptic pathway from moment to moment. In other words, the MSR dynamics likely reflect a high-dimensional process because trial-to-trial fluctuations arise from interactions among multiple physiological sources (e.g., ongoing dorsal horn activity, presynaptic inhibition, and motoneuron excitability). Previous studies have shown that MSR variability in humans, shaped by multiple interacting sources, exhibits scale-invariant (fractal-like) structure and long-range temporal correlations [16,17,18,19]. The MSR therefore occupies a unique dynamical regime: simple enough to permit precise stimulation and measurement, yet sufficiently embedded in the living spinal cord’s network to test the limits of chaos–control techniques in a biologically realistic setting.

This study was designed to address a fundamental gap: whether OGY control can be robustly implemented in vivo, where assumptions of low dimensionality and minimal noise are violated. By applying OGY perturbations to regulate MSR variability in anesthetized, spinalized cats, we sought to determine whether the unstable periodic orbits inferred from MSR return maps could be stabilized despite the pervasive synaptic noise in the intact dorsal horn. Our findings, despite rigorous implementation and verification using canonical chaotic systems, reveal that OGY control fails to reduce MSR variability in vivo. This result, while contrasting with in vitro successes, aligns strikingly with theoretical predictions about noise sensitivity and exposes a crucial boundary condition for chaos control in biological systems.

Understanding these limits is essential not only for basic neuroscience but also for the broader pursuit of dynamical control of neural activity. If classical chaos–control algorithms falter in vivo, new strategies—more robust to high dimensionality, synaptic noise, and network correlations—must be developed before dynamical-systems approaches can be broadly applied to living neural circuits.

## 2. Materials and Methods

### 2.1. General Procedures

All procedures conformed to the Principles of Laboratory Animal Care (NIH Publication No. 85-23, revised 1985) and were approved by the institutional ethics committee of the Benemérita Universidad Autónoma de Puebla (CICUAL-Proyecto-UALVIEP-17/1, 17 November 2017). Six adult cats weighing 3.0–4.5 kg were initially anesthetized with sodium pentobarbital (35 mg/kg, intraperitoneal). Supplemental doses of pentobarbital (10 mg/kg, intravenous) were administered as required to maintain deep anesthesia throughout surgery and data collection. Adequacy of anesthesia was continuously evaluated by verifying stable arterial pressure between 90 and 110 mmHg, fixed and constricted pupils, and the absence of cardiovascular or motor responses to noxious cutaneous stimulation.

After tracheotomy, animals were mechanically ventilated with a Harvard Apparatus respirator. End-tidal CO_2_ was maintained between 3% and 4%, and core temperature was kept at 37 ± 0.2 °C using a servo-controlled heating system. Following completion of the surgical exposure, the animals were paralyzed with pancuronium bromide (0.2 mg/kg/h, intravenous). Anesthesia adequacy was continuously monitored and maintained independently of paralysis.

A laminectomy from L4 to S1 exposed the lumbar spinal cord. Complete spinal transection was performed at the T12 segment using fine surgical scissors, and the lesion site was packed with Gelfoam to prevent reconnection. The absence of descending influences was verified by the loss of hindlimb withdrawal reflexes and by the disappearance of dorsal root potentials evoked by noxious skin stimuli applied rostral to the transection. The left hindlimb was then secured in a stereotaxic frame to minimize movement.

The left gastrocnemius-soleus (GS), posterior biceps-semitendinosus (PBSt), and sural (SU) nerves were dissected free from surrounding tissue, sectioned distally, and mounted on bipolar silver hook electrodes for stimulation. Each nerve was immersed in a pool of warmed mineral oil maintained at 37 °C, ensuring stable impedance and preventing dehydration. The L6 and L7 ventral roots were sectioned distally and reflected onto bipolar silver hook electrodes for extracellular recordings. The dorsal root entry zone was inspected continuously to ensure that no mechanical stress or pressure affected excitability in the monosynaptic pathway.

### 2.2. Stimulation and Recording

Monosynaptic reflexes were elicited by stimulation of group I afferents in the GS or PBSt nerves with constant-current, monophasic square pulses delivered at 0.4 Hz with an AMPI ISO-Flex Stimulus Isolator (A.M.P.I. Jerusalem, Israel) and MASTER-8 pulse generator (A.M.P.I. Jerusalem, Israel). The pulse duration was held constant at 0.1–0.2 ms, and the initial intensity was adjusted to 1.1–1.3 × the threshold required to evoke a clear monosynaptic reflex (MSR) in the L6 or L7 ventral root. During OGY-control trials, stimulus intensity served as the system-wide control parameter and was adjusted on a trial-by-trial basis according to the OGY algorithm. In a complementary set of experiments, the SU nerve served as the control pathway, as its conditioning effects have been shown to reduce MSR variability via presynaptic inhibition.

Afferent volleys were recorded continuously using a bipolar electrode placed across the dorsal root entry zone and a second electrode attached to the paravertebral musculature. Only trials in which the dorsal root volley amplitude varied by less than 2% were accepted for analysis, ensuring that changes in MSR amplitude reflected physiological variability rather than fluctuations in stimulus delivery.

Extracellular MSR responses were amplified using a differential AC amplifier Grass-Astromed P511 (West Warwick, RI, USA) (bandpass 30–3000 Hz; gain 1000–5000) and digitized at a sampling rate of at least 10 kHz with a Digidata 1320A from Axon Instruments, Molecular Devices (San Jose, CA, USA). The amplitude of each MSR, denoted $A_{n}$, was defined as the peak-to-peak value measured within a fixed latency window of 5–12 ms after the stimulus. Trials contaminated by mechanical artifacts, ventilator coupling, amplifier saturation, or abrupt impedance changes were excluded from analysis.

### 2.3. Implementation of the OGY Chaos–Control Algorithm

We have implemented a MATLAB R2024B (MathWorks, Inc., Natick, MA, USA) program based on the theoretical background of experimental chaos control. We have implemented the OGY method to control MSR fluctuations, assuming that unstable periodic orbits can be detected in the return map of the monosynaptic reflex amplitude *A*_*n*+1_ versus the previous amplitude *A_n_*. The successive amplitudes *A_n_* were graphically displayed, and a learning phase was performed to detect the approximate location of the unstable fixed point.

The OGY control algorithm required tuning of two main components: the initial estimate of the unstable fixed point used to define the local neighborhood for Jacobian estimation, and the magnitude constraint placed on the stimulation parameter perturbation. The fixed point was estimated during the learning phase from the MSR return map, and this estimate served as the reference state for defining the neighborhood in which control could be applied. The effective control gain was implemented by limiting the maximum allowable trial-to-trial stimulation perturbation (*δp*_*n*_), ensuring that control inputs remained physiologically small and comparable to normal experimental fluctuations. These tuning steps were applied consistently across all trials and preparations.

**Figure 1:**
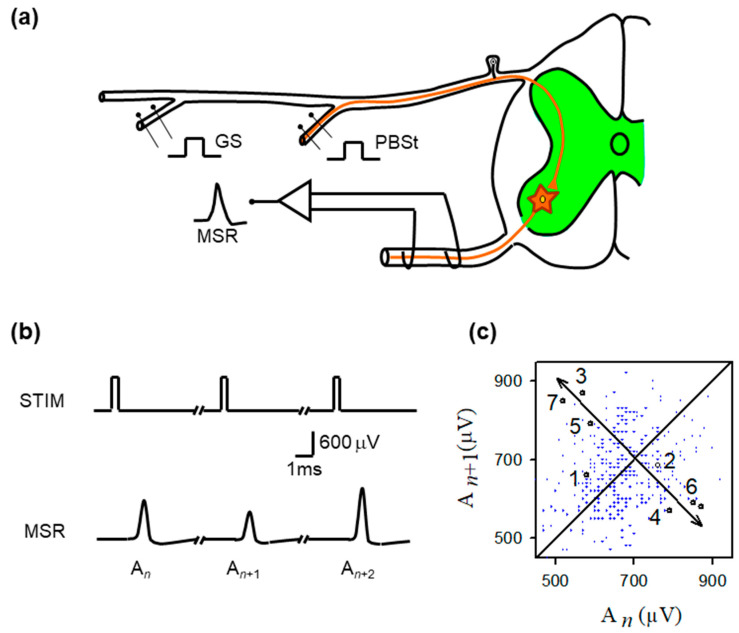
Experimental preparation, monosynaptic reflex recordings, and return-map structure of MSR variability. (**a**) Schematic of the in vivo spinal preparation used to examine monosynaptic reflex (MSR) dynamics. Group I afferents from the gastrocnemius (GS) or posterior biceps–semitendinosus (PBSt) nerves were electrically stimulated while MSRs were recorded extracellularly from the L6–L7 ventral roots. The afferent volley enters the dorsal horn and monosynaptically excites motoneurons (green region). The recorded MSR reflects the aggregate response of the motoneuron population to each identical stimulus pulse. (**b**) Representative sequence of MSRs evoked by constant-intensity GS stimulation. Despite identical stimulation pulses (STIM), successive MSRs exhibit substantial amplitude variability (labeled $A_{n}$, $A_{n+1}$, $A_{n+2}$), illustrating the intrinsic trial-to-trial fluctuations that constitute the target of chaos–control interventions. (**c**) Return map obtained from the MSR amplitudes ($A_{n+1}$ plotted against $A_{n}$). Although small clusters of sequentially ordered points (1–7) transiently approach a common region before diverging, the overall distribution shows broad, irregular scattering, consistent with strong synaptic-driven variability rather than a stable, low-dimensional structure. The diagonal line marks unity; arrows highlight the brief approach-to-divergence trajectory characteristic of an unstable fixed point.

The OGY method uses a linear approximation of the dynamics in the neighborhood of the desired fixed point. The OGY method causes the system-wide parameter to move the stable manifold to the system state point. We selected the intensity of stimulating pulses (applied to GS, PBSt, or SU) as the system-wide parameter, which was adjusted according to the OGY equations. We chose this parameter because the amplitude of monosynaptic reflexes can be readily altered by varying the intensity of stimulating pulses. Figure 1a illustrates the experimental method. All data acquisition and real-time computations were performed in MATLAB R2024B (MathWorks, Inc., Natick, MA, USA). Peak detection was performed using cubic-spline interpolation within the MSR window, and the OGY update loop communicated directly with the AMPI ISO-Flex constant-current isolator to deliver the adjusted stimulus for the subsequent trial.

### 2.4. Statistics

For each experimental condition, the amplitude of consecutive monosynaptic reflexes (MSRs) was quantified by computing the coefficient of variation (CV = SD/mean) across successive responses obtained under constant-strength afferent stimulation. To assess the effect of OGY-based control, CV values recorded before OGY application were compared with those obtained during the control. When the MSR amplitude distributions were not assumed to be normally distributed, all paired comparisons were performed using the Wilcoxon signed-rank test; otherwise, we used a paired *t*-test. This analysis was applied both to experiments in which the stimulation intensity of the GS or PBSt nerves served as the control parameter and to additional experiments in which the stimulation amplitude of the sural (SU) nerve was varied. Data are reported as mean ± SD, and statistical significance was defined as *p* < 0.05. All computations and analyses were performed in MATLAB R2024B (MathWorks, Inc., Natick, MA, USA).

## 3. Results

### 3.1. In Vivo Monosynaptic Reflexes Exhibit Irregular Fluctuations

Figure 1b shows typical monosynaptic reflexes (MSR) with amplitudes (*A_n_*, *A*_*n*+1_, *A*_*n*+2_) produced by stimulation (STIM) of the GS nerve. Figure 1c is the return plot of amplitudes of MSR (*A*_*n*+1_) versus the previous amplitude (*A_n_*). From the observation of the return plot, we found the approximate location of unstable periodic orbits (UPOs), as well as the corresponding location of any fixed point $A_{F}$. To stabilize this fixed point, we next examined all pairs of MSR amplitudes within the approximated fixed-point range threshold (<40 µV).

**Figure 2:**
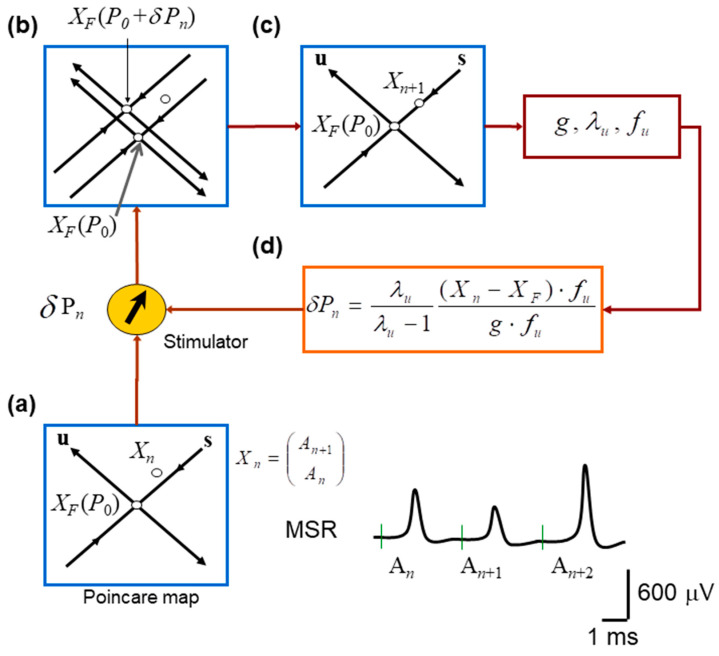
Schematic implementation of the OGY chaos–control algorithm applied to monosynaptic reflex fluctuations. (**a**) Construction of the Poincaré (return) map used to represent the system state on each trial. Consecutive monosynaptic reflex amplitudes form the embedded point $X_{n}$ in a two-dimensional state space, with the estimated unstable fixed point $X_{F}$ located at the intersection of the inferred stable (s) and unstable (u) manifolds. (**b**) Effect of introducing a small perturbation in stimulation intensity. A slight change in the control parameter shifts the system state relative to the manifolds of $X_{F}$, revealing the local geometry of the dynamics and the direction of divergence along the unstable manifold. (**c**) Illustration of the OGY corrective step: using the estimated eigenstructure of the map, the next state $X_{n+1}$ is nudged toward the stable manifold of the fixed point, thereby attempting to counteract the natural tendency of trajectories to diverge. (**d**) Overview of the real-time control loop. The OGY algorithm computes the required perturbation for each trial and delivers it to the nerve stimulator, which adjusts the amplitude of the next afferent stimulus to stabilize the reflex response.

Specifically, the unstable fixed point was identified empirically from the MSR return map $\left(A_{n+1}\;\mathrm{vs}.\;A_{n}\right)$ by locating the region where short sequences of points transiently approach the identity line $A_{n+1}=A_{n}$ before diverging. This brief approach-and-diverge behavior is consistent with the local dynamics near an unstable fixed point. In this geometric interpretation, the fixed point corresponds to the intersection of the locally inferred stable and unstable manifolds, as illustrated schematically in Figure 2.

Because the in vivo spinal preparation exhibits substantial trial-to-trial physiological variability, the return map lacks a sharply defined low-dimensional attractor, and the fixed point cannot be localized to a single precise coordinate. Therefore, the fixed-point location $X_{F}$ (and corresponding scalar amplitude $A_{F}$ was treated as an approximate region estimated during the learning phase described in the Methods.

Pairs of amplitudes of MSR were defined as the embedded return-map state vectors

$$X_{n}=\left[\begin{array}{c} A_{n+1}\\ A_{n} \end{array}\right],X_{n+1}=\left[\begin{array}{c} A_{n+2}\\ A_{n+1} \end{array}\right],X_{F}(p_{0})=\left[\begin{array}{c} A_{F}\\ A_{F} \end{array}\right].$$

Because the OGY method relies on a local linear approximation of the dynamics, only points sufficiently close to the estimated unstable fixed point were retained. Specifically, we defined a fixed-point neighborhood (also referred to as the “time-point neighborhood”) as the set of embedded points

$$S=\left\{X_{n}:∥X_{n}-X_{F}(p_{0})∥<r\right\},$$

where *r* is a radius defining the admissible neighborhood size around the fixed point. In practice, this criterion corresponded to selecting return-map points whose MSR amplitudes were within the approximated fixed-point range threshold (typically <40 µV) so that (i) the system was close enough to the fixed point for local linearization to remain valid, and (ii) enough points were available to estimate the Jacobian matrix reliably. This threshold is expected to influence control performance because it determines the size of the fixed-point neighborhood used for local linearization: if the threshold is too small, too few points are available to estimate the local Jacobian and eigenstructure reliably, whereas if it is too large, included points may fall outside the region where the local linear approximation required by OGY remains valid.

Within this neighborhood, the return-map dynamics were approximated by the local linear model

$$X_{n+1}-X_{F}(p_{0})=M\;[X_{n}-X_{F}(p_{0})],X_{n}\in S,$$

where *M* is the estimated local Jacobian matrix. From *M*, we calculated the stable and unstable eigenvalues $\left(\lambda_{s},\lambda_{u}\right)$, eigenvectors $\left(e_{s},e_{u}\right)$, and contravariant eigenvectors $\left(f_{s},f_{u}\right)$.

Next, we slightly changed the stimulation amplitude from $p_{0}+\delta p$, and computed the fixed-point sensitivity vector

$$g={\frac{\partial X_{F}(p)}{\partial p}|}_{p=p_{0}}.$$

We attempted to control the oscillations in the amplitude of the monosynaptic reflex when

$$∥X_{n}-X_{F}(p_{0})∥<\left(\frac{\lambda_{u}-1}{\lambda_{u}}\right)\delta p^{*}\;(g\cdot f_{u}),$$

and the OGY perturbation was defined as

$$\delta p_{n}=\frac{\lambda_{u}}{\lambda_{u}-1}\;\frac{(X_{n}-X_{F}(p_{0}))\cdot f_{u}}{g\cdot f_{u}}.$$

### 3.2. Local Linearization and Real-Time OGY Control Were Successfully Implemented

Figure 2 illustrates the procedure for controlling MSR fluctuations. Note that the stimulation amplitude was successively adjusted according to the equations obtained from the OGY method. Figure 1b illustrates a sequence of monosynaptic reflexes (MSR) with amplitudes *A_n_*, *A*_*n*+1,_ and *A*_*n*+2_ evoked by a sequence of afferent stimuli of constant strength (STIM). Figure 1c displays a typical return map obtained from the monosynaptic reflex amplitude *A*_*n*+1_ versus the previous amplitude *A_n_*.

Using the OGY method (Figure 2), control of monosynaptic reflex variability was attempted in six anesthetized cats. In these experiments, the amplitude of stimulation to the gastrocnemius plus soleus (GS) or posterior biceps and semitendinosus (PBSt) nerves was varied to control monosynaptic reflex fluctuations. These operations confirm that real-time state estimation, parameter adjustment, and stimulation delivery were functioning as intended in the in vivo preparation.

To assess the robustness of OGY performance to tuning, we examined how small variations in the estimated fixed-point location and in the allowable stimulation perturbation range influenced control activation. As expected, shifting the fixed-point estimate or adjusting the perturbation constraint changed the number of return-map points classified within the control neighborhood, thereby altering how frequently the controller was engaged. However, across reasonable choices of fixed-point initialization and perturbation constraints, OGY control did not consistently reduce MSR variability compared with baseline conditions. In contrast, the same real-time OGY implementation stabilized the canonical Hénon map (Section 3.5), suggesting that the lack of control in vivo was not due to incorrect implementation but rather reflects limitations imposed by physiological variability and complex spinal network dynamics.

### 3.3. OGY Perturbations Fail to Stabilize MSR Amplitude Fluctuations

**Figure 3:**
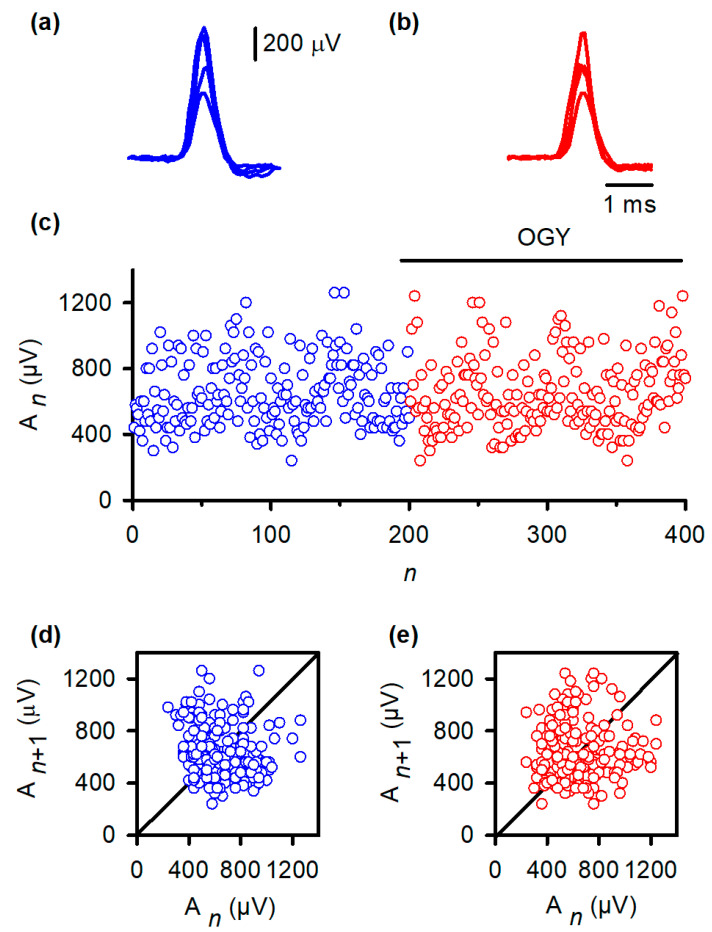
OGY control fails to stabilize monosynaptic reflex fluctuations in vivo. (**a**) Superimposed monosynaptic reflex (MSR) responses recorded during constant-strength stimulation of the gastrocnemius nerve. Despite identical afferent input, reflex amplitudes vary substantially from trial to trial, illustrating the intrinsic variability of the in vivo pathway. (**b**) MSR responses obtained while the OGY control algorithm continuously adjusted stimulation intensity. The reflexes remain highly variable, demonstrating that state-dependent perturbations do not suppress amplitude fluctuations. (**c**) Trial-wise MSR amplitudes before (blue) and during (red) application of OGY control. Although the algorithm actively perturbs the stimulation parameter, the amplitude distribution remains broad and unstable, with no discernible reduction in variability. (**d**) Return map of baseline responses ($A_{n+1}$ vs. $A_{n}$). Points form a widely scattered cloud, reflecting the high-dimensional, synaptic-driven variability inherent to the spinal MSR circuit. (**e**) Return map during OGY control. The distribution remains similarly diffuse, indicating that OGY perturbations do not collapse trajectories toward a fixed point or uncover stabilizable low-dimensional structure.

Despite accurate algorithmic implementation, OGY control did not reduce MSR variability in any of the six animals studied. Representative MSR traces recorded during control (Figure 3b) remained as variable as baseline responses (Figure 3a). When amplitudes were plotted across trials, both conditions produced similarly broad envelopes (Figure 3c). Quantitatively, the coefficient of variation (CV) did not change significantly with OGY intervention (baseline: 0.26 ± 0.11; OGY: 0.31 ± 0.11; Student’s *t*-test, t = 0.65, df = 10, *p* = 0.52).

Return maps reinforced this conclusion. Under baseline conditions (Figure 3d), the relationship between *A_n_* and *A*_*n*+1_ formed a widely dispersed cloud, indicating substantial intrinsic variability. During OGY control (Figure 3e), the map remained virtually identical: points did not collapse toward the fixed point, nor did the system exhibit any reduction in the spread of trajectories. These results indicate that, although correctly directed by the OGY rules, the perturbations were ineffective at overcoming the strong endogenous fluctuations that dominate the MSR pathway in vivo.

### 3.4. Presynaptic Modulation via Sural Nerve Stimulation Does Not Enhance Control Efficacy

Because conditioning volleys in the sural nerve (SU) can reduce MSR variability by increasing presynaptic inhibition, we tested whether SU stimulation amplitude could serve as a more physiologically effective control parameter. However, this alternative intervention also failed to stabilize the reflex pathway. Variability remained unchanged (baseline CV: 0.15 ± 0.08; OGY CV: 0.17 ± 0.09; Wilcoxon signed-rank test, W = 8.5, *p* = 0.84), mirroring the lack of efficacy observed when GS or PBSt stimulation intensity served as the control parameter. Thus, even physiologically relevant modulatory pathways could not support OGY stabilization under in vivo conditions.

### 3.5. Successful Stabilization of the Hénon Map Confirms Correct Implementation of the OGY Algorithm

**Figure 4:**
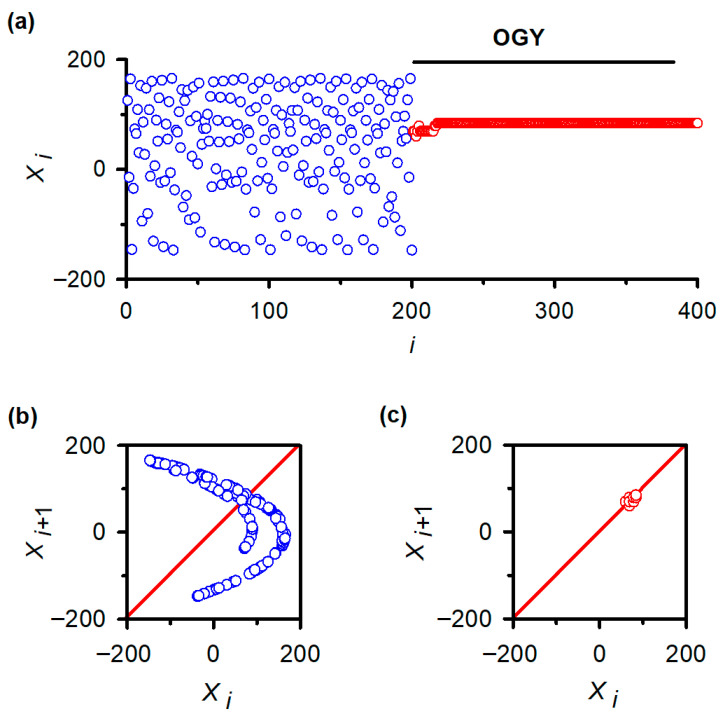
Validation of OGY chaos control in a canonical low-dimensional system: the Hénon map. (**a**) Iterative behavior of the Hénon map before and after activation of the OGY control algorithm. Prior to control (blue points), the variable $x_{i}$ displays the characteristic irregular fluctuations of chaotic dynamics. When the OGY controller is engaged at approximately iteration 200, the trajectory (red points) rapidly converges toward the unstable fixed point and remains stably localized thereafter, demonstrating successful stabilization in a system known to contain accessible unstable periodic orbits. (**b**) Return map of the uncontrolled Hénon sequence ($x_{i+1}$ vs. $x_{i}$). The broad, folded structure reflects the geometry of the chaotic attractor. The diagonal line marks the identity axis used to identify fixed points. (**c**) Return map during OGY control. Points collapse into a compact cluster surrounding the fixed point, indicating that the perturbations effectively stabilize the previously unstable orbit. This positive control confirms the correctness of the algorithmic implementation used in the in vivo experiments.

To verify that the negative results in vivo were not due to implementation errors, we applied the same MATLAB routines to the classical Hénon map. As expected, the uncontrolled Hénon sequence displayed hallmark chaotic fluctuations (Figure 4a, blue trace), and its return map exhibited the characteristic folded attractor (Figure 4b). After activating the OGY controller at iteration ~200, the sequence rapidly converged onto the unstable fixed point (Figure 4a, red trace) and remained stably localized for the remainder of the simulation. The corresponding return map collapsed from a broad chaotic set into a tight cluster surrounding the fixed point (Figure 4c), replicating canonical demonstrations of OGY control in deterministic low-dimensional systems.

This verification establishes that the algorithm, numerical routines, and stimulation-control logic were functioning correctly. The failure of OGY to stabilize reflex fluctuations, therefore, reflects intrinsic properties of the in vivo spinal circuitry rather than a computational or experimental artifact.

Across all animals, control parameters, and analytic approaches, OGY perturbations were unable to reduce MSR variability or reveal hidden periodic structure in the spinal reflex pathway. These results demonstrate that, despite occasional UPO-like motifs in the return map, the full in vivo MSR circuitry operates in a regime in which endogenous synaptic activity and network noise overwhelm the fine-scale geometric structures on which OGY relies for stabilization.

## 4. Discussion

### 4.1. Failure of OGY Control In Vivo Reveals Fundamental Constraints Imposed by Spinal Network Dynamics

The present study demonstrates that the OGY chaos–control method is unable to stabilize fluctuations in monosynaptic reflexes (MSRs) in the anesthetized, spinalized cat. This inability to impose control in vivo contrasts sharply with the successes reported in physical systems [3], isolated cardiac tissue [5], hippocampal slice preparations [6], and even stochastic neuronal models [7]. The divergence between these positive demonstrations and our negative findings underscores a central insight: the intact spinal cord violates core assumptions required for OGY control, particularly those concerning dimensionality and noise.

OGY control depends on the presence of accessible unstable periodic orbits (UPOs) embedded within a low-dimensional attractor. Our experimental return maps showed neither the dense-orbit structure nor the local geometric regularity required for reliable stabilization. Instead, trial-to-trial MSR fluctuations reflected large, irregular modulations incompatible with the small parametric deviations assumed in classical chaos control. The failure of OGY in this context, therefore, aligns precisely with the theory articulated by Ott, Grebogi, and Yorke: effective stabilization becomes impossible when noise overwhelms the local structure surrounding a UPO [2].

### 4.2. Synaptic-Driven Fluctuations Dominate MSR Variability In Vivo

Our interpretation aligns with extensive physiological evidence showing that MSR amplitude variations arise from ongoing synaptic bombardment within the dorsal horn and motoneuronal pools. Spontaneous dorsal horn activity contributes strongly to the negative cord dorsum potentials that correlate with MSR amplitude variations [20]. Similarly, intracellular recordings reveal that motoneuron membrane potential is dynamically sculpted by postsynaptic excitatory and inhibitory inputs during the reflex cycle [21]. These synaptic-driven fluctuations represent high-dimensional physiological noise, not the small, deterministic parameter variations required by the OGY algorithm.

Thus, rather than revealing a hidden chaotic structure, MSR variability in vivo reflects continuous, distributed synaptic input that destabilizes any fluctuation patterns. Under these conditions, the OGY method cannot anchor the system to a fixed point, because the system rarely—if ever—occupies the narrow neighborhood where the algorithm’s linear approximation holds.

This perspective motivates quantitative approaches to assess the effective dimensionality of MSR fluctuations, for example, by estimating their fractal or correlation dimension, as in previous studies [16,17,18,19]. Although we did not compute an explicit dimensionality estimate for the present MSR recordings, the experimental findings still support our central conclusion: OGY control does not reliably reduce MSR variability in vivo. Importantly, we confirmed that our real-time implementation was functioning correctly by demonstrating successful stabilization of the canonical low-dimensional Hénon map.

### 4.3. Why OGY Succeeds In Vitro but Fails In Vivo

The discrepancy between our negative in vivo findings and prior in vitro successes can be understood through two essential distinctions. First, in vitro preparations such as cardiac monolayers and hippocampal slices dramatically reduce the dimensionality of the active network. With fewer active neurons or cells, the underlying attractor becomes low-dimensional, enabling the reliable identification of UPOs and their associated stable and unstable manifolds. Second, noise levels in vitro are tightly constrained, allowing the system to remain sufficiently close to a target orbit for OGY perturbations to take effect.

In contrast, the in vivo spinal cord receives dense convergent sensory inflow and exhibits substantial intrinsic spontaneous activity. Its continuous synaptic dynamics push the system into a high-dimensional regime where fixed points are difficult to identify and nearly impossible to stabilize reliably. These properties make the spinal cord an inhospitable environment for classical chaos–control techniques that depend on precise local linearization.

### 4.4. Computational Theories of Correlated Noise Provide a Mechanistic Explanation

Recent computational studies provide a conceptual bridge between our empirical findings and theoretical expectations [22]. Engelken et al. showed that in balanced recurrent networks, correlated inputs, analogous to the shared synaptic drive impinging on motoneurons, can severely impede attempts to suppress ongoing chaotic variability [23]. Their dynamic mean-field analysis reveals that recurrent circuitry cancels out or counteracts common input signals, rendering external control efforts ineffective.

Our results mirror this prediction. The MSR pathway receives strong common synaptic input from dorsal horn circuits, which likely overwhelms the subtle, trial-specific perturbations generated by the OGY algorithm. In this sense, our in vivo findings extend the theoretical predictions of Engelken and colleagues into a biological network: correlated synaptic noise eliminates the dynamical foothold required for chaos control.

### 4.5. Limitations

A limitation may be that stimulation amplitude is a relatively weak, indirect global control parameter for regulating MSR variability in the intact spinal cord. Although stimulus intensity can reliably change the mean size of the monosynaptic response, trial-to-trial fluctuations are strongly shaped by intrinsic spinal mechanisms, such as ongoing dorsal horn synaptic activity, presynaptic inhibition, and dynamic changes in motoneuron excitability, which can vary independently of the stimulus amplitude. Under these conditions, small parameter perturbations applied at the peripheral nerve may not produce a sufficiently consistent shift of the system state toward the stable manifold of the targeted fixed point, even when the OGY algorithm is implemented correctly. Thus, the failure of OGY control in vivo may reflect not only noise and effective high dimensionality, but also a mismatch between the control parameter accessible experimentally (stimulation amplitude) and the internal physiological variables that dominate the moment-to-moment state of the spinal reflex pathway.

Other limitations of our study should be considered. Although we manipulated two control parameters—stimulus intensity to GS/PBSt afferents and to the SU nerve—other physiological parameters such as neuromodulatory tone, presynaptic release probability, or motoneuron membrane excitability were not directly accessible. The OGY method also requires precise, real-time localization of the system state near a UPO, a challenge posed by physiological noise and limited recording resolution. Additionally, our experiments were conducted under acute anesthesia and spinal transection, conditions that may alter the intrinsic dimensionality of spinal circuits.

Although we interpret the failure of OGY control as being limited by physiological noise and effective high dimensionality, we did not attempt an exhaustive quantification of “noise levels” using the broad range of possible metrics available in neural time-series and nonlinear-dynamics analysis. For example, variability can be summarized using amplitude dispersion measures (e.g., SD, variance, CV, IQR), return-map dispersion metrics (e.g., distance to the estimated fixed point, neighborhood occupancy rate, or local linearization residuals), temporal correlation measures (e.g., autocorrelation structure, DFA/Hurst exponent), or nonlinear complexity metrics (e.g., correlation dimension, Lyapunov exponents, entropy-based measures). Performing a systematic analysis across these complementary measures would substantially expand the methodological scope of the present study. Importantly, however, our main conclusion does not depend on selecting any single noise metric: OGY control did not reliably reduce MSR variability as quantified by CV, and the return-map structure remained broadly dispersed under control. At the same time, the same real-time implementation successfully stabilized the low-dimensional Hénon map.

From a broader physical perspective, intact neural circuits can be viewed as a form of active biological matter, in which internal activity, ongoing interactions, and continuous state fluctuations dominate the dynamics. This differs from many “hard-matter” experimental systems where chaos–control methods are commonly validated under relatively stable, low-noise conditions. In this context, our results suggest that OGY control, designed to exploit a reproducible low-dimensional geometric structure, may not generalize well to living spinal circuitry, where the effective state is continuously reshaped by synaptic bombardment and physiological variability. This limitation is consistent with recent discussions of the challenges and opportunities of applying control concepts in active/living matter systems [24].

### 4.6. Future Directions

Given the limitations of OGY control in noisy, high-dimensional biological environments, future work should evaluate strategies designed to tolerate or harness biological noise. One promising alternative is delayed feedback control [25], which stabilizes periodic orbits without requiring explicit identification of fixed points. Adaptive chaos–control methods may also prove more robust by adjusting control parameters in real time [26].

The occasional bang–bang (OBB) method represents another promising avenue [27]. Rather than applying continuously graded perturbations, OBB uses discrete, fixed-magnitude interventions triggered only when certain dynamical conditions are met. Such coarse, discontinuous control may outperform finely tuned perturbations in the presence of physiological noise.

Experimental manipulations that reduce background synaptic activity, such as selective deafferentation, pharmacological modulation of inhibitory circuits, or reduction of neuromodulatory drive, may reveal threshold conditions under which chaos–control becomes feasible. Moreover, quantifying the fractal dimension of MSR dynamics could clarify how intrinsic noise elevates the system’s dimensionality and identify regimes in which low-dimensional attractors transiently emerge [28].

Future work could strengthen the mechanistic link between physiological variability and control failure by incorporating explicit quantitative metrics of noise and variability directly relevant to OGY performance in vivo. This could include estimating return-map neighborhood occupancy (i.e., how frequently the system state enters the fixed-point neighborhood required for local linearization), measuring the mean distance of trajectories from the fixed point and from the identity line, and evaluating local linearization residuals as a proxy for the effective stochastic drive that disrupts orbit tracking. In parallel, characterizing the temporal correlation structure of MSR fluctuations (e.g., via autocorrelation/DFA methods) and applying nonlinear complexity estimates (e.g., correlation dimension or entropy-based metrics) could help identify whether low-dimensional structure emerges transiently under specific physiological conditions. Such analyses may reveal regimes in which classical chaos–control methods become partially viable or guide the design of control strategies that are explicitly robust to correlated physiological noise and high-dimensional network dynamics.

Future work could apply surrogate-based randomness tests and higher-dimensional embedding analyses to further the extent to which MSR variability reflects stochastic fluctuations rather than low-dimensional deterministic structure.

### 4.7. Alternative Chaos–Control Strategies and Implications for the Regulation of MSR Fluctuations

The OGY method stabilizes unstable periodic orbits by applying small parameter perturbations only when the system is in a narrow neighborhood where a local linear approximation is valid. This requirement makes OGY highly sensitive to strong physiological variability and unstable return-map geometry, which likely explains its failure to reduce MSR variability in vivo despite correct implementation.

A widely used alternative is Pyragas time-delayed feedback control, which uses delayed versions of the measured signal to promote the stabilization of periodic behavior and can be non-invasive once stabilization is achieved [25].

Unlike OGY, Pyragas control does not require explicit fixed-point identification or estimation of stable/unstable manifolds, which may offer practical advantages in noisy biological data. Reviews of chaos control highlight Pyragas delayed feedback as a major complementary strategy to OGY, with different requirements for measurement and tuning [29].

Other approaches focus on robustness to noise and uncertainty, including adaptive and sliding-mode–based strategies that improve stability guarantees under disturbances [30]. However, these approaches often assume richer access to state information or model structure than is typically available in MSR experiments, where only reflex amplitude is observed, and stimulation amplitude is the primary controllable variable.

More recently, data-driven control methods, including reinforcement learning approaches, have been proposed to stabilize chaotic systems while avoiding explicit local model estimation, and have been tested on standard chaotic maps [31]. While translation to real-time physiology requires careful safety constraints, such methods may ultimately provide alternatives for systems dominated by high-dimensional physiological variability.

Overall, these comparisons reinforce our conclusion that OGY fails in vivo not because of implementation error (as validated on the Henon map), but because MSR fluctuations rarely remain within the stable low-dimensional neighborhood required for reliable local control. Future MSR studies may therefore benefit most from exploring Pyragas-type delayed feedback as a realistic next step, as well as from more robust or adaptive strategies explicitly designed for noisy biological systems.

## 5. Conclusions

We conclude that, unlike the non-noisy Henon map, the real-time implementation of the OGY method failed to reduce MSR amplitude variability in the in vivo cat spinal cord. Our findings substantiate a central theoretical prediction of Ott, Grebogi, and Yorke: when noise dominates system dynamics, classical chaos–control strategies fail. In the in vivo spinal cord, MSR variability reflects dense, ongoing synaptic bombardment and high-dimensional neuronal interactions, rendering the OGY method ineffective despite its success in vitro and in non-biological systems. These results define a crucial boundary for the applicability of low-dimensional chaos–control algorithms in intact neural circuits and underscore the need for new, noise-tolerant control strategies that better match the complexity of biological systems. Developing such approaches will be essential for translating dynamical systems theory into practical tools for regulating neural activity in vivo.

## Data Availability

The original contributions presented in this study are included in the article. Further enquiries can be directed to the corresponding author.

## Abbreviations

The following abbreviations are used in this manuscript:

MSR	Monosynaptic Reflex
OGY	Ott–Grebogi–Yorke (chaos–control method)
UPO	Unstable Periodic Orbit
GS	Gastrocnemius–Soleus nerve
PBSt	Posterior Biceps–Semitendinosus nerve
SU	Sural nerve
CV	Coefficient of Variation
SD	Standard Deviation
CA3	Cornu Ammonis area 3 (hippocampus)
MATLAB	Matrix Laboratory (computing environment)
